# Mother-Baby Day Hospital (MBDH): preliminary results of effectiveness of multidisciplinary intensive intervention for women with postpartum affective/anxiety disorder

**DOI:** 10.1192/j.eurpsy.2022.843

**Published:** 2022-09-01

**Authors:** E. Gelabert, A. Torres Giménez, S. Andrés-Perpiñá, C. Naranjo, E. Roda, L. Garcia-Esteve, A. Roca Lecumberri

**Affiliations:** 1 Universitat Autònoma de Barcelona, Psicologia Clínica I De La Salut, Bellaterra (Barcelona), Spain; 2 Hospital Clínic de Barcelona, Servei De Psiquiatria I Psicologia. Unitat De Salut Mental Perinatal, Barcelona, Spain

**Keywords:** Mother-Baby Day Hospital, Perinatal anxiety disorders, Perinatal mood disorders, Perinatal interventions

## Abstract

**Introduction:**

Women experiencing postpartum mental illness have unique needs. Psychiatric Mother Baby Units (MBUs) can provide specialist in-patient care for mothers without separation from their baby. Since 2018, an innovative Mother-Baby Day Hospital (MBDH) have been developed and implemented in a public hospital in Spain, directed at the intensive, integral, and multidisciplinary treatment.

**Objectives:**

The aim of the present study was to obtain preliminary data regarding its effectiveness in postpartum women with affective and anxiety disorders.

**Methods:**

Thirty-three mothers and their babies with affective or anxiety disorders attended to MBDH CLINIC-BCN participated in the study. All women were assessed at admission, discharge, and 3 months follow-up. Primary outcomes were depression (EPDS) and anxiety symptoms (STAI-S), mother-infant bonding (PBQ) and functional impairment (HoNOs).

**Results:**

At discharge, 100% of women no longer met the full criteria for the main diagnosis (PSR≥5). Significant improvements from admission to discharge were achieved in depression and anxiety symptoms, mother infant bonding and functional impairment. Clinical significance was also calculated. After treatment, mothers had greater autonomy for care their babies. Similar results were observed at 3 months follow-up. The MBDH was rated by mothers as an excellent quality program and they would recommend it.

**Conclusions:**

This study found that multidisciplinary intervention at MBDH for postpartum women with affective or anxiety disorders is effective, not only for maternal psychopathology but also for maternal care and bonding. It is imperative to develop specialized devices that integrate the care of the dyad by professionals specialized in perinatal mental health.

**Disclosure:**

No significant relationships.

